# PGI_2_ as a Regulator of Inflammatory Diseases

**DOI:** 10.1155/2012/926968

**Published:** 2012-07-18

**Authors:** Stacy L. Dorris, R. Stokes Peebles

**Affiliations:** Division of Allergy, Pulmonary, and Critical Care Medicine, Vanderbilt University School of Medicine, T-1218 MCN, Nashville, TN 37232-2650, USA

## Abstract

Prostacyclin, or PGI_2_, is an end product derived from the sequential metabolism of arachidonic acid via cyclooxygenase and PGI synthase (PGIS). The receptor for PGI_2_, IP, can be found on a variety of cell types and signaling through this receptor exhibits broad physiological effects. Historically, PGI_2_ has been understood to play a role in cardiovascular health, specifically having powerful vasodilatory effects via relaxation of smooth muscle and inhibiting of platelet aggregation. For these reasons, PGI_2_ has a long history of use for the treatment of pulmonary arterial hypertension (PAH). Only recently, its importance as an immunomodulatory agent has been investigated. PGI_2_ regulates both the innate and adaptive immune systems and its effects are, for the most part, thought to be anti-inflammatory or immunosuppressive in nature, which may have implications for its further clinical use.

## 1. Introduction

Prostacyclin, or PGI_2_, was first reported by Needleman and Vane in 1976 and is an end product derived from the sequential metabolism of arachidonic acid via cyclooxygenase-2 (COX-2) and prostacyclin synthase (PGIS) [1]. COX-2 is expressed upon specific stimulation such as cytokines, growth factors, bacterial endotoxins, tumor promoters, and hormones by macrophages, neutrophils, and activated mesenchymal cells [2–4]. There is rare expression of COX-2 in unstimulated tissues [5–7], but it can be present at low basal levels in endothelium and the renal macula densa [2, 5]. COX-2 is typically associated with proinflammatory conditions such as atherosclerotic lesions, aortic aneurysms, or vascular damage where COX-2 derived products likely provide a protective effect [8–10]. COX-2 is inhibited by nonsteroidal anti-inflammatory (NSAIDS) and specific COX-2 inhibitors, which may have tissue specific effects.

Several additional cells types have been shown to express COX-2 and PGIS and they include fibroblasts, follicular dendritic cells, endothelial cells, smooth muscle cells, and thymic nurse cells. Production of PGI_2_ is decreased by the inhibition of PGIS by tyrosine-nitrating agents such as peroxynitrite [11] and tetranitromethane [12]. Lastly, PGIS can be limited by substrate-dependent suicide inactivation if there is adequate conversion of PGH_2_, the substrate for PGIS, which causes accumulation of inactivated enzyme [13].

PGI_2_ is primarily produced in mammalian vasculature with elevated levels in pulmonary arterial segments when compared to systemic circulation [14]. As such, PGI_2_ has been understood to play a role in cardiovascular health specifically inhibiting platelet aggregation and having powerful vasodilatory effects via relaxation of smooth muscle [5, 15]. PGI_2_ analogues have been successfully used for therapy in pulmonary arterial hypertension, peripheral occlusive disease, vascular complication of diabetes mellitus, and treatment of reperfusion injury. Only recently, its importance as an immunomodulatory agent has been investigated.

## 2. PGI_2_ Receptor Signaling

**Figure 1:**
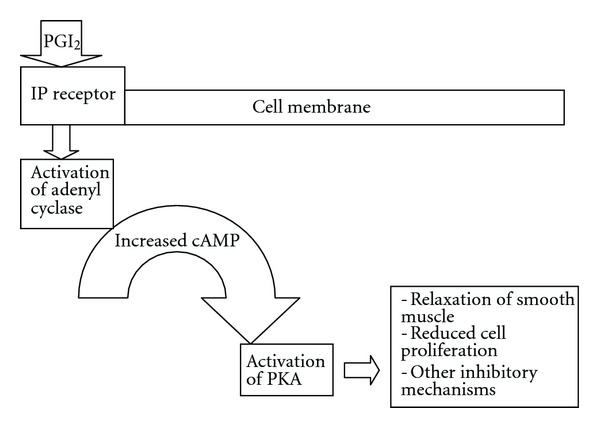
PGI_2_ receptor signaling.

The cell surface receptor for PGI_2_ is a seven transmembrane G-protein-coupled receptor termed IP [6]. IP is coupled to a guanosine nucleotide-binding *α*-stimulatory protein (G*α*_s_). When activated by PGI_2_, IP stimulates adenyl cyclase leading to increased intracellular cyclic AMP (cAMP) (see Figure 1). Increased cAMP then leads to activation of protein kinase A (PKA) and further phosphorylation of key proteins [14–16]. These actions culminate in relaxation of smooth muscle, reduced cell proliferation, and other inhibitory mechanisms. IP is found on a variety of cell types and exhibits broad physiological effects. Mouse IP receptors have been identified on neurons, smooth muscle cells of the aorta, coronary arteries, pulmonary arteries, and megakaryocytes. Human IP receptors are present on multiple cell types including platelets, medullary thymocytes, neutrophils, dendritic cells, eosinophils, T regulatory cells, and activated T cells [4, 17]. IP receptors are also found on many cell types in the lung such as macrophages, pneumocytes, smooth muscle cells, and fibroblasts [17]. In addition to the single known membrane IP receptor, a peroxisome proliferator-activated nuclear receptor (PPAR) functions as a transcription factor after activation by PGI_2_ [3, 15]. There are three PPAR isoforms, *α*, *δ*  (*β*), and *γ* [15]. PPAR*γ*, expressed in adipose tissue, spleen, and large intestines predominately, is thought to be downstream of the activated IP membrane receptor and can be stimulated via stable PGI_2_ analogues. PPARs are responsive to not only PGI_2_ but also a broad range of ligands [15].

## 3. PGI_2_ in Specific Disease States

### 3.1. Allergic Inflammation

One of the areas that PGI_2_ has been extensively studied is allergic inflammation. PGI_2_ is produced in the human lung during allergic reactions, suggesting that it may have a regulatory role in allergen-induced inflammation [18, 19]. The *in vivo* role of PGI_2_ in mediating allergic inflammation has been investigated both in IP-deficient mice, which examines the role of endogenous PGI_2_ signaling, as well as exogenous administration of PGI_2_. First, we will review studies that use IP-deficient mice in determining how endogenous PGI_2_ modulates allergen-induced lung disease.

In an acute model of allergic inflammation induced by ovalbumin (OVA), IP-deficient mice had significantly greater airway inflammatory responses consisting of increased plasma extravasation, leukocyte accumulation, and both IL-4 and IL-5 production in the airways after sensitization and exposure to inhaled antigen [20]. In addition, the IP-deficient mice had elevated total serum IgE and antigen-specific IgE when compared to wild-type mice [20]. These findings support endogenous PGI_2_ as an important suppressor of acute allergic inflammation. Similar findings were reported in a chronic model of OVA-induced lung inflammation. In this IP-deficient mouse model, there was a significant increase in the level of inflammatory leukocytes in the airway, serum OVA-specific IgE, and lung expression of T helper type 2 (Th2) cytokines such as IL-4, IL-5, and IL-13. The IP-deficient mice had more subepithelial fibrosis compared to wild-type mice, possibly related to the upregulation of collagen synthesis [21]. Therefore, inability to signal through IP led to enhanced acute and chronic allergic inflammation and airway remodeling.

In addition to IP-deficient mice, the role of COX-2 inhibitors contributes to the knowledge of how endogenous PGI_2_ regulates allergic inflammation. After OVA inhalation in a DO11.10 transgenic mousemodel of T-cell-mediated airway inflammation, there was an increased level of PGI_2_ [4]. Blocking PGI_2_ via COX-2 inhibition resulted in a marked increase in Th2 mediated lung inflammation in response to OVA challenge. COX-2 inhibition and the prevention of PGI_2_ formation was associated with increased bronchial airway hyperresponsiveness, elevated lung expression of Th2 cytokines, and decreased IL-10 production [4]. These results highlight a possible risk enhanced airway inflammation with use of specific COX-2 inhibitors in allergic asthmatics by blocking production of an immunoinhibitory prostanoid such as PGI_2_.

Animal models of exogenous PGI_2_ administration further supports that this prostanoid inhibits allergic inflammation. In an OVA sensitized mouse model using adoptive transfer of DO11.10 Th2 cells pretreated with PGI_2_, there was significantly decreased pulmonary inflammation and airway hyperreactivity [22]. A protective effect of PGI_2_ on acute airway function is further suggested by a null effect on ozone induced airway inflammation and hyperresponsiveness in mPGES-1 deficient mice. PGI_2_ metabolites in the BAL of these mice were increased, while changes in other prostanoids favored deleterious effects on lung function [23]. PGI_2_ suppressed Th2 infiltration of the lung via strong inhibition of CCL17-induced chemotaxis. IP deficient Th2 cells were unaffected and migrated normally [22]. Lastly, in a mouse model of asthma, inhaled iloprost, a PGI_2_ analogue, decreased the cardinal features of asthma such as Th2 cytokine production, eosinophilic airway inflammation, goblet cell hyperplasia, and bronchial airway hyperresponsiveness [24]. Iloprost inhibited the maturation and migration of antigen presenting lung myeloid dendritic cells, decreased costimulatory molecules, and decreased the induction of allergen-specific Th2 response [24]. These findings suggest a role for inhaled iloprost in the treatment of asthma.

### 3.2. Inflammation-Induced Anorexia

We have shown that PGI_2_ signaling via the IP receptor reduces allergic inflammation. The effect of decreased appetite in acute inflammation, such as in the setting of IL-1*β* and lipopolysaccharide (LPS) administration, has been suggested to be PG dependent. In a mouse model, PGI_2_ signaling decreased the level of circulating ghrelin, a peptide produced predominantly by the stomach, which has potent stimulatory effects on appetite [25]. This finding was very similar to the effect of LPS on circulating levels of ghrelin and suggests a role for PGI_2_ in the acute sickness behavior of anorexia. IL-1*β* induced ghrelin expressing cells to produce PGI_2_. Nonspecific inhibition of PG production via NSAIDs reversed the decrease in circulating ghrelin caused by the acute inflammatory stimulation of LPS specifically [25]. These results suggest that PGI_2_ may suppress appetite in certain acute inflammatory disease states.

### 3.3. Liver Injury

In a mouse model of a concanavalin-A (ConA-) induced immune-mediated liver injury mimicking hepatic inflammation, beraprost, a PGI_2_ analogue, decreased tissue damage [7]. COX-2 deficient mice developed more severe ConA-induced liver damage compared to wild-type mice or COX-1 deficient mice. Treatment with beraprost/ConA had a protective effect with a more than 10-fold decrease in serum alanine aminotransferase (ALT) levels compared to those treated with vehicle/ConA alone. Hepatic mRNA levels and expression of both TNF-*α* and IFN-*γ* by natural killer T cells (NKT) and T cells, key in the development of ConA induced liver disease, were decreased in the COX-2 deficient mice after treatment with beraprost when compared to vehicle/ConA alone treated mice. The protection provided by beraprost is postulated to stem from the maintenance of hepatic blood flow via beraprost's vasodilatory effects [7]. These findings suggest that PGI_2_ analogues may be of benefit to patients suffering from inflammatory liver disease such as hepatitis due to viral infection, autoimmune conditions, use of certain drugs, or alcohol ingestion.

### 3.4. Cardiovascular Disease

It is hypothesized that early depletion of PGI_2_ from endothelial tissue could lead to the pathogenesis of atherosclerosis by causing deposition of adipocyte lipid in smooth muscle cells [26]. Cellular micro RNA (miRNA) is an important known negative regulator of gene expression. PGI_2_ regulated miRNA expression in a mouse adipose tissue-derived cell line leading to diminished deposition of lipid in cells [15]. The importance of this lies in the possibility of a relationship between loss of normal PGI_2_ production in the setting of obesity and atherosclerosis.

Iloprost was investigated for its effects on leukocyte adherence in intestinal venules and subsequent microvascular blood flow in a rat endotoxemia model. Iloprost attenuated leukocyte adherence in both postcapillary and collecting intestinal venules and improved intestinal microvascular blood flow without affecting mean arterial pressure or heart rate [27]. These findings suggest a role for iloprost therapy to reduce endotoxin-induced intestinal injury.

Retinoic acid induces PGIS and therefore synthesis of PGI_2_ in human umbilical vein endothelial cells [2]. Retinoic acid is important in the development of the cardiovascular system during embryonic development, angiogenesis and has antithrombotic and antiatherogenic qualities. 13-*cis*-retinoic acid (13-*cis*-RA) a molecule with anti-inflammatory, antitumor, and immunomodulatory effects elevated PGI_2_ levels as measured by 6-oxo-PGF_1*α*_. Consistently, arachidonic acid induced platelet aggregation was inhibited by 13-*cis*-RA. Treatment with the inflammatory cytokine IL-1*β* alone rapidly inactivated PGIS in these same cells followed by a diminution of PGI_2_. These findings support a role for 13-*cis*-RA as a possible selective treatment for patients with inflammatory cardiac disease. When IL-1*β* was given in combination with 13-*cis*-RA, 13-*cis*-RA was able to overcome the inhibitory effects of IL-1*β*, leading to increased PGIS expression and PGI_2_ levels [2]. Similarly, in human vascular smooth muscle cells exposed to IL-1*β*, hypoxia overcame the inhibitory effects of IL-1*β* and increased PGI_2_ production [5]. Hypoxia alone in these same cells elevated PGIS expression and PGI_2_ levels. These findings suggest that hypoxia could drive an adaptive response in vascular cells and plays a role in protecting vascular cells when inflammation is present [5].

### 3.5. Emphysema

PGIS expression was lower in arteriolar endothelium of human emphysema lung tissue compared with normal lung [28]. Cigarette smoke extract suppressed PGIS gene expression suggesting that its decrease could lead to deleterious effects on lung vasculature in this setting. Mice exhibiting overexpression of PGIS in the pulmonary vasculature had decreased endothelial cell apoptosis after chronic tobacco smoke exposure [28]. Cigarette smoke extract may bind to CpG sites in DNA leading to disruption of transcriptional regulation suggesting a mechanism for the minimization of PGIS gene expression.

### 3.6. Cytokine-Mediated Inflammation

IL-1*β*, TGF-*β*, and bradykinin, in human pulmonary artery smooth muscle, decreased the production of cAMP in response to subsequent administration of PGI_2_ analogues, providing data that inflammation can impair the actions of PGI_2_ analogues in pulmonary hypertension treatment [14]. IL-1*β*, TGF-*β*, and bradykinin also decreased adenylyl cyclase mRNA and increased G-*α* inhibitory (G*α*_i_) protein levels with subsequent reduction in IP mRNA expression [14]. Importantly, this could be a rational for the development of tolerance to PGI_2_ analogue medications.

Cytokine toxicity is mediated in many cell types through the arachidonic acid metabolism pathway via inducible COX-2 production [3]. Proinflammatory cytokines are major effectors of programmed cell death in the development of type 1 diabetes mellitus. Using a model of human insulin producing pancreatic *β* cells, PGIS overexpression protected against cytokine toxicity via decreased activation of the transcription factor nuclear factor kappa-light-chain-enhancer of activated B cells (NF-*κ*B) and subsequent prevention of inducible nitric oxide synthase (iNOS) and decreased cytokine-induced caspase-3 activation. Therefore, low-endogenous PGIS expression may have a pathogenic part in the development of pancreatic *β* cell death [3].

### 3.7. Fibrosis

PGI_2_ has regulatory affects on fibroblast proliferation. For example, in a bleomycin-induced pulmonary fibrosis mouse model, PGI_2_ functioned as an antiproliferative molecule preventing an increase in fibroblasts and providing protection against loss of lung function [17]. Similarly, in an additional mouse model of bleomycin-induced pulmonary fibrosis, mice treated with iloprost had a significant reduction in airway and pulmonary parenchyma inflammatory cells and deposition of collagen with improvement in lung static compliance, tissue elastance, and overall survival [29]. The proposed mechanism for these changes is the upregulation of antifibrotic mediators such as interferon-*γ* (IFN-*γ*), the chemokine CXCL10, and the downregulation of proinflammatory and profibrotic cytokines such as TNF-*α*, IL-6, and TGF-*β* [29].

Using a mouse model of PGIS overexpression specifically in lung epithelium, mortality related to bleomycin-induced acute lung injury was decreased [30]. In addition to decreased mortality, there was a reduction in parenchymal consolidation, apoptosis of lung tissue and weight loss. These findings were explained by *in vitro* and *in vivo* PGI_2_ induced expression of NADP(H) : quinone oxide reductase 1 (Nqo1), an enzyme known to inhibit the generation of reactive oxygen species and therefore protective against oxidative stress. The PGIS-overexpressing mice had elevated levels of this antioxidant prior to administration of bleomycin and afterwards [30]. Taken together, these three studies suggest that PGI_2_ may provide protection against bleomycin-induced lung injury.

### 3.8. Viral Infection

Signaling through IP had protective effects in the setting of respiratory syncytial virus (RSV) infection in a mouse model and one study suggests it may be beneficial in human disease. During RSV infection, in a mouse model of overexpressed PGIS in bronchial epithelium, weight loss, viral replication, and IFN-*γ* production were all decreased compared to controls [31]. Histopathology results showed decreased pulmonary edema. In contrast, IP-deficient mice had longer more severe illness with prolonged viral replication [31].

RSV infection also elevated a urinary metabolite of PGI_2_ in human infants [32] suggesting PGI_2_ may modulate virally-induced illness in people. Low numbers of a genetic polymorphism in the PGIS gene with subsequent decreased urinary PGI_2_ metabolite levels, described as a genetic 9-base variable-number tandem repeat (VNTR), were correlated with more severe RSV infection. Infants with greater VNTR had less severe RSV-induced infections. Therefore, an association between lower numbers of PGIS VNTR repeats and increased severity of RSV infection identifies a host genetic factor associated with RSV disease severity [32]. Both of these studies support a protective role for PGI_2_ in RSV-induced illness and suggest a possible therapy for acute RSV infection.

### 3.9. Rheumatoid Arthritis

In contrast to the seemingly anti-inflammatory properties of PGI_2_, there is still debate about its effect in the setting of specific conditions [16, 33, 34]. PGI_2_ and PGE_2_ have been questioned as causative factors in inflammation as levels are increased in inflammatory tissues. The synovial fluid of rheumatoid arthritis (RA) patients has rich quantities of PGI_2_ but the exact role of PGI_2_ in RA is not fully understood [33, 34]. In a mouse model of chronic RA, IP-deficient mice were subjected to collagen-induced arthritis and had significantly decreased clinical and histologic arthritic scores despite anticollagen antibodies and complement activation similar to wild-type mice [34]. These mice were noted to have decreased levels of IL-6 in their arthritic paws. Administration of an IP agonist elevated the inflammatory cytokine IL-6 and amplified arthritis related genes such as IL-11, VEGF, FGF-2, and RANKL, increasing inflammation in the joint. Elevated IL-6 led to proliferation of the synovium, maturation of B cells, and formation of osteoclasts, all important in the pathogenesis of RA [34]. Importantly, signaling through the IP receptor required the presence of inflammatory IL-1*β* for many of its effects in RA. One mechanism proposed for the effects of increased PGI_2_ was the enhanced production of IL-6 via activated synovial fibroblasts in the setting of IL-1*β*. Lack of elevated IL-6 due to IL-1*β* explains why patients receiving PGI_2_ agonists, for example, due to PAH, do not exhibit an increase in arthritic symptoms as a side effect. The results of this study are intriguing because PGI_2_ was traditionally thought to play a role in the mediation of acute inflammation, but its role in chronic inflammation has been less studied [34].

 Similarly, in an IP-deficient mouse model of collagen-antibody induced chronic inflammatory arthritis, the reduction in arthritis scores was 91% compared to a wild-type control group [33]. When a highly selective IP antagonist was used in a RA mouse model, it decreased pain similar to use of NSAIDs [33]. Therefore, it appears that signaling through the IP receptor, in the setting of RA, may increase inflammation in the joint. Currently, it is believed that both PGI_2_ and PGE_2_ are the primary prostaglandins involved in the inflammatory pain response in RA and this provides a rationale for the empiric use of NSAIDS and COX inhibitors for treatment of chronic arthritis.

## 4. PGI_2_ Regulation of the Immune System

### 4.1. PGI_2_ Regulation of the Innate Immune System

PGI_2_ regulates both innate and adaptive immunity and its effects are, for the most part, anti-inflammatory or immunosuppressive in nature. PGI_2_ modulates the function of dendritic cells, macrophages, monocytes, endothelial cells and eosinophils [35–41]. We will now review this data.

Dendritic cells are an important bridge between the innate and the adaptive immune system. PGI_2_ analogues decreased mouse bone marrow derived dendritic cell (BMDC) maturation, function, and proinflammatory cytokine production after LPS stimulation. PGI_2_ analogues also increased production of anti-inflammatory IL-10 and diminished chemokine production in mice suggesting an overall anti-inflammatory effect [42]. In a dose-dependent fashion, PGI_2_ analogues, decreased secretion of TNF-*α*, IL-1*α*, IL-6, and IL-12 *in vitro*. After LPS stimulation, both iloprost and cicaprost decreased levels of costimulatory molecules CD86, CD40, and MHC class II molecules on BMDC. The BMDC had a diminished ability to activate antigen-specific CD4 T cells of DO11.10 mice and led to reduced IL-4 and IL-5 production by the CD4 cells after iloprost and cicaprost treatment [42]. When the biological activity of iloprost on human monocytes-derived dendritic cells was examined, similar findings were identified. In a dose dependent fashion, iloprost inhibited the secretion of TNF-*α*, IL-6, IL-8, and IL-12 by monocytes-derived dendritic cells and increase secretion of IL-10 [39].

PGI_2_ analogues produced a differential pattern of regulation on alveolar versus peritoneal rat macrophages suggesting variation in immunomodulatory effects of these agents on these specific cell types. Using a well characterized FcR-mediated model of phagocytosis, activation of the IP receptor inhibited phagocytosis of IgG-opsonized targets in peritoneal macrophages to a much greater extent compared to alveolar macrophages [35]. In addition, under conditions of LPS treatment, peritoneal macrophages increased production of IL-6 after administration of iloprost or carbaprostacyclin, but alveolar macrophages did not increase production to the same degree. Iloprost and carbaprostacyclin also significantly inhibited peritoneal macrophage of bacterial killing when compared to alveolar macrophages. A postulated reason for the differences is the differential expression of IP receptors on the two macrophage cell types and different receptor binding properties of PGI_2_ analogues [35].

In addition to its effects on mouse innate immune cells, PGI_2_ also has important regulatory effects on human dendritic cells. Iloprost increased IL-10 expression but inhibited toll-like receptor-mediated expression of TNF-*α* and IFN-*α* in human plasmacytoid dendritic cells suggesting an overall anti-inflammatory role [36]. Iloprost may, therefore, increase the tolerogenic ability of plasmacytoid dendritic cells and could potentially be useful in asthma treatment. Human follicular dendritic cells, found in germinal centers of secondary lymphoid follicles, strongly express PGIS and are able to produce PGI_2_. Application of beraprost significantly reduced T cell proliferation stimulated by anti-CD3 antibody and, therefore, production of PGI_2_ in the germinal centers may be a mechanism for controlling T cell numbers [38] suggesting a reason why T cells constitute a smaller population when compared to B cells in the germinal centers. Iloprost reduced IFN-*γ* and IL-6 induced MCP-1, IL-8, RANTES, and TNF-*α* production in human monocytes [40]. STAT1 activation, critical in cardiovascular inflammation, was reduced with iloprost administration and led to decreased IFN-*γ* induced MCP-1 expression and therefore iloprost had an overall anti-inflammatory effect [40].

The evaluation of PGI_2_ analogues on the expression of Th1 and Th2 related chemokines has also been ongoing as chemokines are known to play a role in the development of asthma [43]. Monocytes are main contributors of chemokines. Human monocytes were pretreated with iloprost and treprostinil prior to LPS stimulation and then evaluated for production of Th1-related chemokines interferon-*γ*-inducible protein-10 (IP-10/CXCL10) and Th2-related chemokine macrophage-derived chemokine (MDC/CCL22). The PGI_2_ analogues decreased IP-10 production, but enhanced MDC. The enhancement of MDC suggests that use of PGI_2_ analogues could potentially increase Th2 inflammation [43].

Lastly, in a model using human lung microvascular endothelial cells, eosinophils and the chemoattractants eotaxin and C5a, PGI_2_ inhibited eosinophilic migration through the endothelial barrier by affecting their chemotaxis, adhesion, and transmigration and by strengthening the endothelial barrier [37]. Chemotaxis was limited by direct stimulation of the IP receptor on eosinophils as PGI_2_ caused upregulation of cAMP leading to an increase is adenylyl cyclase despite use of chemoattractants such as eotaxin and C5a. Rapid upregulation of eotaxin-induced CD11b adhesion molecule was diminished by PGI_2_ leading to decreased adhesion to fibronectin. PGI_2_ also prevented eosinophil transmigration by strengthening endothelial barrier function. These findings were reversed by exposing eosinophils and endothelium to an IP antagonist [37]. These studies are important because they suggest a role for PGI_2_ in the maintenance of the endothelial barrier and the prevention of allergic disease.

### 4.2. PGI_2_ Regulation of the Adaptive Immune System

Currently, it is widely believed that signaling through the IP receptor has immunosuppressive properties. PGI_2_ analogues play a key role via inhibition of Th1 and Th2 cytokine production from CD4 T cells [21, 42] although the specifics remain controversial as we will see in the discussion below. B cells are also influenced by analogues of PGI_2_ and are discussed at the end of this section.

In a mouse study using PGI_2_ analogues, cicaprost and iloprost, IFN-*γ* from Th1 cells and IL-4, L-10, and IL-13 from Th2 cells were diminished in a dose dependent manner [41]. Elevated cAMP levels and downregulation of NF-*κ*B correlated with the inhibition of these cytokines. These findings suggest an immunosuppressive capability of PGI_2_ analogues [41]. In IP-deficient mice subjected to a chronic contact hypersensitivity model showed a significantly decreased contact hypersensitivity response [44]. In contrast to the study above, iloprost promoted Th1 cells differentiation suggesting that PGI_2_-IP-signaling promotes contact hypersensitivity. Promotion of Th1 differentiation was decreased by a PKA inhibitor suggesting mediation through the cAMP-PKA pathway [44]. These findings suggest a role for PGI_2_ in the promotion of Th1 pathology, which is in opposition to findings suggesting that it has more immunosuppressive effects.

In addition to immunomodulatory effects on T cells, PGI_2_ regulates B cell function. In activated B cells, beraprost increased the costimulatory molecule CD86 via the IP receptor with subsequent elevation in cAMP [45]. CD86 is important as it acts as a costimulatory molecule required for T cell activation specifically Th2 activation. Its expression is elevated on B cells in the light zone of germinal center where some Th2 cells are found. Increased T cell proliferation was noted after exposure to beraprost treated B cells [45] and suggests a role for beraprost as an adjuvant in vaccine development.

## 5. Therapeutic Use of PGI_2_

PGI_2_ has a long history of use for the treatment of pulmonary arterial hypertension (PAH) [14, 16, 28]. Regrettably, it efficacy has been less than expected possibly due to the development of tolerance. A postulated mechanism for the development of tolerance is the impaired production of PGI_2_ in the setting of increased thromboxane synthesis, which is also thought to be an initial step in the pathogenesis of PAH. Nonetheless, it is one of the main therapies in severe idiopathic PAH and has been shown to improve overall survival.

Epoprostenol, treprostinil, and iloprost are approved for the treatment of PAH [46] (see Table 1). Their use in PAH has been investigated since 1980 and their initial use in long-term therapy began in 1984 in the research setting. Intravenous formulations are nonselective and can cause both pulmonary and systemic hypotension via vasodilation. Side effects shared by these medications include headache, flushing, hypotension, jaw pain with initial mastication, diarrhea, nausea, musculoskeletal pain of the legs and feet, and an erythematous blotchy skin rash [46].

Epoprostenol (Flolan), a stable freeze dried synthetic salt of PGI_2_, is also used in transplantation, renal dialysis, and extracorporeal circulation systems as it is an effective inhibitor of platelet aggregation. It was the first prostanoid approved for use in the treatment in PAH by the FDA in 1995 and is currently the most commonly used PGI_2_ analogue [46]. It has a very short half life of 6 minutes *in vivo*. Any interruption of its infusion can cause severe rebound pulmonary hypertension and even death. Tolerance may develop over time. Due to its unstable nature requiring a continuous intravascular infusion, its use as an antithrombotic in the general population has been limited.

Treprostinil (Remodulin) was approved for use by the FDA in 2002 and is a potent IP receptor agonist. It is administered as a continuous subcutaneous (SQ) or IV infusion or an inhaled formulation and provides a long-term survival benefit in patients with idiopathic PAH. Pain at the site of administration when given SQ has limited this form of administration. The inhaled form given 4 times per day was approved for use in 2009. Currently, an oral form of treprostinil is being investigated. Overall, treprostinil has fewer side effects when compared to epoprostenol [46] and a longer half life of about 4 hours.

Iloprost (Ventavis), a PGI_2_ analogue and potent IP receptor agonist, received FDA approval in 2004. It is administered via nebulization 6–9 times per day requiring 10–15 minutes per administration. Its half life is about 20–30 minutes. Administration via inhalation limits side effects such as systemic hypotension. Inhaled iloprost can cause the development of reactive airway obstruction therefore limiting its use [46].

Cicaprost is a synthetic PGI_2_ analogue which is metabolically stable and bioavailable after oral administration. It is currently used in the research setting [46].

Beraprost is a stable, orally active PGI_2_ analogue, used experimentally in the treatment of primary pulmonary hypertension, peripheral occlusive disease, ischemia, reperfusion injury, and in the vascular complication of diabetes mellitus [7]. Beraprost has a high-affinity binding to the human IP receptor and a longer half life of about 1 hour [45]. Benefits of beraprost have been shown in short-term trials but it appears to have attenuated effects in longer treatment courses [46].

### 5.1. Potential New Therapeutic Options

ONO-1301 is a novel nonprostanoid long-acting PGI_2_ agonist that is currently being used in the research setting. It has both PGI_2_ activity and thromboxane synthase inhibitory activity [47]. It is unlike PGI_2_ as it does not contain a five-membered ring and allylic alcohol. This property adds to its stability and allows the drug to be given two times per day as a subcutaneous injection. In mouse studies, it diminished pulmonary fibrosis associated with bleomycin intratracheal injection and improved survival rates. In addition, it decreased the total cell count, neutrophil count, thromboxane B2, and total protein level in BAL fluid and inhibit ICAM-1 and VCAM-1 adhesion molecule expression in the lung tissue [47]. Therefore, it may be a prospective candidate in the treatment of idiopathic pulmonary fibrosis in the future.

Taken together, PGI_2_ functions mostly as an immunoinhibitor molecule through multiple cell types such as dendritic cells, macrophages, and T-cells.

## Figures and Tables

**Table 1:** Therapeutic use of PGI_2_—approved agents.

Agent	Pharmacology	Indications
Epoprostenol	Synthetic salt of PGI_2_	PAH, transplantation, renal dialysis, and extracorporeal circulation systems
Treprostinil	IP receptor agonist	PAH
Iloprost	IP receptor agonist	PAH

## Abbreviations

ALT: Alanine aminotransferase
BMDC: Bone marrow dendritic cells
CCL17: Chemokine (C-C motif) ligand 17
13-*cis*-RA: 13-*cis*-retinoic acid
CD: Clusters of differentiation
ConA: Concanavalin A
CXCL10: C-X-C motif chemokine 10
cAMP: Cyclic adenosine monophosphate
COX: Cyclooxygenase
FGF-2: Fibroblast growth factor
FDA: Food and Drug Administration
G*α*_i_: Guanosine nucleotide-binding-*α*-inhibitory protein
G*α*_s_: Guanosine nucleotide-binding *α*-stimulatory protein
iNOS: Inducible nitric oxidesynthase
IgE: Immunoglobulin-E
IFN-*γ*: Interferon-*γ*
IL: Interleukin
LPS: Lipopolysaccharide
MDC: Macrophage derived chemokine
MHC: Major histocompatibility complex
mRNA: Messenger RNA
miRNA: Micro-RNA
MCP-1: Monocyte chemotactic protein-1
mPGES-1: Membrane-associated PGE synthase-1
Nqo 1: NADP(H) : Quinone oxide reductase
NKT: Natural killer T cells
NSAIDS: Nonsteroidal anti-inflammatory
NF-*κ*B: Nuclear factor kappa-light-chain-enhancer of activated B cells
OA: Osteoarthritis
OVA: Ovalbumin
PPAR: Peroxisome proliferators activated receptor
IP: Prostacyclin receptor
PGIS: Prostacyclin synthase
PGI_2_: Prostacyclin
PG: Prostaglandin
PKA: Protein kinase A
PAH: Pulmonary arterial hypertension
RANKL: Receptor activator of nuclear factor kappa-B ligand
RANTES: Regulated upon activation, normal Tcell expressed, and secreted protein
RSV: Respiratory syncytial virus
RA: Rheumatoid arthritis
STAT: Signal transducers and activators of transcription
SQ: Subcutaneous
Th1: T helper type 1 T cells
Th2: T helper type 2 T cells
TGF-*β*: Transforming growth factor-*β*
TNF-*α*: Tumor necrosis factor-*α*
VNTR: Variable-number tandem repeat
VEGF: Vascular endothelial growth factor.
